# A Study on the Clinical Profiles of Patients With Cerebrovascular Accident (Stroke) in a Tertiary Care Hospital in Jharkhand

**DOI:** 10.7759/cureus.35919

**Published:** 2023-03-09

**Authors:** Madhusudan Kumar, Abhay Kumar, Usha Saroj, Ravi Kumar, Satyendra K Singh, Anil K Choudhary, Zobia Farheen, Shimpy Priya

**Affiliations:** 1 Internal Medicine, Medinirai Medical College, Daltonganj, IND; 2 General Medicine, Rajendra Institute of Medical Sciences, Ranchi, IND; 3 Pathology, Rajendra Institute of Medical Sciences, Ranchi, IND; 4 General Medicine, Mahatma Gandhi Memorial Medical College, Jamshedpur, IND

**Keywords:** anemia, renal dysfunction, proteinuria, diabetes mellitus, hypertension, stroke

## Abstract

Introduction

Stroke is a devastating and disabling cerebrovascular disease with a significant amount of residual deficit. The prevalence of stroke is in a rising trend in India. Larger studies are needed for the evaluation of risk factors.

Material and methods

This cross-sectional study aimed to assess the clinical profile of patients with stroke. The demographic details of the patients were taken, comorbidities were noted, and laboratory tests were done.

Observation

The most common age group who presented with stroke was 61-80 years, followed by 41-60 years, comprising 47% and 46%, respectively. Ischemic stroke was more common (60%) than hemorrhagic stroke (40%). Male patients were more than female patients. Alcohol, smoking, hypertension, diabetes, anemia, and proteinuria were present in the study group.

Conclusion

Regular evaluation of blood pressure, blood sugar, lipid profile, and proteinuria should be routinely done in patients with diabetes and hypertension who are more than 40 years old.

## Introduction

Cerebrovascular accidents (stroke) constitute a global health epidemic and a leading cause of sustained disability and mortality. William Cole likely first introduced the word “stroke” into medicine in 1689 in A Physico-Medical Essay Concerning the Late Frequencies of Apoplexies [1]. A transient ischemic attack is defined as a transient episode of neurological dysfunction caused by focal brain and spinal cord or retinal ischemia, without acute infarction [2]. Cerebrovascular accidents (stroke) are of two types: hemorrhagic stroke and ischemic stroke. The risk of stroke is associated with increased age, previous stroke or transient ischemic attack, hypertension, smoking, diabetes mellitus, hypercholesterolemia, and atrial fibrillation/flutter [3]. Abnormalities of blood glucose and altered lipid profiles are often associated with stroke and should be taken into consideration for better secondary prevention [4]. Transient ischemic attacks provide an opportunity to prevent strokes that physicians encounter. Transient ischemic attacks should be treated as a medical emergency, as up to 80% of strokes after transient ischemic attacks are preventable [5]. Hypertension is the most important modifiable risk factor for stroke. Appropriate reduction of blood pressure is necessary for stroke prevention, even more important than the choice of antihypertensive drugs. Lifestyle factors that have been proven to decrease stroke risk include reducing salt intake, ceasing smoking, performing regular physical activity, and maintaining a normal body weight [6].

Age has been the strongest risk factor for both ischemic and hemorrhagic stroke, and its incidence doubles with each successive decade after the age of 55 years. However, there is a considerable portion of patients with significant cerebrovascular disease but without any of these stroke risk factors, leading to the hypothesis that there may be other factors that have not been identified yet that may improve diagnosis and treatment strategies and reduce the related public health burden [7].

Stroke is a heterogeneous disease with multiple additive risk factors and causes. Primary prevention of stroke is related to risk factor modification, and it helps in the significant reduction of the burden of stroke in an aging population. Secondary prevention of recurrent strokes focuses mainly on the workup and tailored management targeted at the mechanisms responsible for the stroke or transient ischemic attack [8]. Hypertension, systolic blood pressure, in particular, is the most common risk factor for stroke in young patients. Diabetes and hypertension are the risk factors for middle-aged patients, while hypertension, diabetes, dyslipidemia, and alcohol consumption are the risk factors for aged patients [9].

## Materials and methods

This was a cross-sectional observational study conducted in a tertiary care hospital. Patients who were admitted with clinical and radiological diagnosis (non-contrast computed tomography (CT) of the brain) of cerebrovascular accident were taken for study. A total number of 100 patients were taken according to inclusion and exclusion criteria. The study duration was 12 months (from December 2020 to November 2021). The definition of stroke has been taken from the World Health Organization definition of stroke as “rapidly developed clinical signs of focal (or global) disturbance of cerebral function, lasting more than 24 hours or leading to death, with no apparent cause other than of vascular origin” [10].

Inclusion criteria

Patients who were more than 18 years with clinically and radiologically confirmed diagnoses of stroke admitted were taken in this study, as well as all patients who are hemodynamically stable and not requiring ventilatory support. We also included only those patients/guardians who gave their consent (Table 1).

**Table 1:** Inclusion criteria

Inclusion criteria	
Age (years)	>18
Diagnosis criteria	Clinical and radiological
Hemodynamic status	Stable
Consent status	Those who gave consent

Exclusion criteria

Patients below 18 years or pregnant females, patients who presented with a transient ischemic attack, and patients with strokes due to trauma were not included in this study (Table 2).

**Table 2:** Exclusion criteria

Exclusion criteria	
Age (years)	<18
Pregnancy status	Pregnant
Hemodynamic status	Unstable
Cause of stroke	Trauma
Type of stroke	Transient ischemic attacks

Patients admitted with clinical and radiologic diagnoses of stroke who fulfilled the inclusion and exclusion criteria were taken into the study after taking their consent. Patients were clinically examined. Medical history about comorbidities was taken in all patients to look for a history of hypertension, diabetes, lipid disorders, etc. Demographic information, such as the age of the patient, the sex of the patient, and any history of alcoholism or smoking, were noted. Laboratory investigations were sent to the laboratory on the first day of the admission of the patient.

These data were recorded in a predesigned proforma. All data were then inserted in an Excel sheet (Microsoft Corporation, Redmond, WA, USA), and all calculations were done in it. All patients were provided treatments according to diagnosis. The study was conducted after obtaining approval from the institutional ethics committee of Rajendra Institute of Medical Sciences (RIMS) (approval number: IEC-236).

## Results

The study aimed to assess the clinical profile of patients with cerebrovascular accidents (stroke) and their risk factors. In this study, 100 patients with stroke who fulfilled the inclusion and exclusion criteria were selected and assessed during their stay in the hospital. The patients included in this study were of age 18 years or above. The most common age group who presented with stroke was 61-80 years, followed by 41-60 years, comprising 47% and 46%, respectively.

Table 3 shows the distribution of sociodemographic risk factors for stroke in both males and females. Of 100 patients, 64 (64%) were male and 36 (36%) were female. Out of 100 patients, 60 (60%) had ischemic stroke and 40 (40%) had hemorrhagic stroke. Out of 60 ischemic stroke patients, 36 (60%) were male and 24 (40%) were female. Out of 40 hemorrhagic stroke patients, 28 (70%) were males and 12 (30%) were females.

**Table 3:** Sociodemographic risk factors

Sociodemographic risk factors		Ischemic stroke (n=60)	Hemorrhagic stroke (n=40)
Sex	Male	36 (60%)	28 (70%)
	Female	24 (40%)	12 (30%)
Age	≤60 years	28 (46.7%)	22 (55%)
	>60 years	32 (53.3%)	18 (45%)
Alcohol		5 (8.3%)	9 (17.5%)
Smoking		8 (13.3%)	6 (15%)

Out of 40 patients with hemorrhagic stroke, 37 were of intraparenchymal hemorrhage and three were of subarachnoid hemorrhage. Out of 60 patients with ischemic stroke, 54 were due to a vascular cause, two were due to tubercular meningitis, and four were due to atrial fibrillation. Out of 100 patients, 48 (48%) were known cases of hypertension. Out of 60 ischemic stroke patients, 24 (40%) had hypertension. Out of 40 hemorrhagic stroke patients, 24 (60%) had hypertension. Out of 48 patients, 24 had hemorrhagic stroke and 24 had ischemic stroke.

Among 100 patients with stroke, 39 (39%) were in the prediabetic range and 35 (35%) were in the diabetic range. Out of 60 ischemic stroke patients, 24 (40%) had diabetes. Out of 40 hemorrhagic stroke patients, nine (22.5%) had diabetes. In the case of ischemic stroke, out of 36 male patients, 12 (33.3%) were in the prediabetic range and 18 (50%) were in the diabetic range. Out of 24 female patients with ischemic stroke, seven (29.1%) were in the prediabetic range and eight (33.3%) were in the diabetic range. In the case of hemorrhagic stroke, out of 28 male patients, 13 (46.4%) were in the prediabetic range and seven (25%) were in the diabetic range. Out of 12 female patients with hemorrhagic stroke, seven (58.3%) were in the prediabetic range and two (16.7%) were in the diabetic range.

The mean hemoglobin level was 11.9 g/dL with a standard deviation of 2.14 g/dL. In ischemic stroke patients, out of 60 patients, 46 (76.7%) had anemia. Out of these 46 patients, 31 were male and 15 were female. In hemorrhagic stroke patients, out of 40 patients, 17 (42.5%) had anemia (Table 4). Out of these 17 patients, 10 were male and seven were female. The mean platelet count was 2.12 lack/mm³ with a standard deviation of 0.97. Out of 60 ischemic stroke patients, 17 (28.33%) had decreased levels of platelet count (eight males and nine females). Out of 40 hemorrhagic stroke patients, 12 (30%) had decreased platelet count (seven males and five females).

**Table 4:** Medical risk factors CAD: coronary artery disease

Risk factors	Ischemic stroke (n=60)	Hemorrhagic stroke (n=40)
Diabetes	24 (40%)	9 (22.5%)
Hypertension	24 (40%)	24 (60%)
Atrial fibrillation	4 (6.7%)	0 (0%)
Dyslipidemia	3 (5%)	1 (2.5%)
CAD	2 (3.3%)	0 (0%)
Tubercular meningitis	2 (3.3%)	0 (0%)
Anemia	46 (76.7%)	17 (42.5%)
Proteinuria	19 (31.7%)	20 (50%)

Table 5 shows that out of 60 ischemic stroke patients, 12 had stage 1 kidney disease, 25 had stage 2 kidney disease, 12 had stage 3A kidney disease, nine had stage 3B kidney disease, none had stage 4 kidney disease, and two had end-stage renal disease (ESRD). Out of 40 hemorrhagic stroke patients, eight had stage 1 kidney disease, 15 had stage 2 kidney disease, 12 had stage 3A kidney disease, two had stage 3B kidney disease, three had stage 4 kidney disease, and nine had ESRD. Out of 60 patients with ischemic stroke, 19 had proteinuria, one had trace proteinuria, 12 had proteinuria of 1+, and six had proteinuria of 2+. Out of 40 patients with hemorrhagic stroke, 20 had proteinuria, one had trace proteinuria, 10 had proteinuria of 1+, and nine had proteinuria of 2+.

**Table 5:** Stages of kidney disease (according to eGFR) Stage 1: healthy kidneys or kidney damage with normal or high GFR, eGFR > 90 mL/minute/1.73 m^2^, stage 2: eGFR of 60-89 mL/minute/1.73 m^2^, stage 3A: eGFR of 45-59 mL/minute/1.73 m^2^, stage 3B: eGFR of 30-44 mL/minute/1.73 m^2^, stage 4: eGFR of 15-29 mL/minute/1.73 m^2^, stage 5: eGFR < 15 mL/minute/1.73 m^2^ eGFR: estimated glomerular filtration rate

Stage	Ischemic stroke (n=60)	Hemorrhagic stroke (n=40)
Stage 1	12 (20%)	8 (20%)
Stage 2	25 (42%)	15 (37.5%)
Stage 3A	12 (20%)	12 (30%)
Stage 3B	9 (15%)	2 (5%)
Stage 4	0 (0%)	3 (7.5%)
Stage 5	2 (3.3%)	0 (0%)

## Discussion

Age is an important factor as most of the patients who presented with stroke were between 61 and 80 years with male preponderance. Ischemic stroke was more common (60%) than hemorrhagic stroke (40%).

A meta-analysis conducted by Zhang et al. suggested that low-to-moderate alcohol intake significantly reduces the risk of total stroke and stroke mortality, but on the other hand, heavy alcohol intake significantly increases the risk of total stroke [11]. In a meta-analysis done by Pan et al., a relationship has been shown between stroke of any type and current smoking status irrespective of gender, and they also found that there is no association between former smokers and the incidence of stroke; this indicated that cessation of smoking has a positive effect on the incidence of stroke [12]. Atrial fibrillation and flutter allow blood to stagnate, particularly in the left atrial appendage, which leads to thrombus formation and subsequent embolization to the cerebral or systemic circulation. The risk of cardioembolic ischemic stroke is high in both permanent and paroxysmal atrial fibrillation [13]. Risk factors such as alcohol, smoking, atrial fibrillation, and coronary artery disease were present in some cases in this study.

Hypertension is the single most important modifiable risk factor for stroke; hypertension has been related to more than half of all strokes worldwide [14]. The American Diabetes Association recommends assessment of fasting blood sugar, glucose tolerance, or A1C level in all at-risk patients and those older than 45 years for screening of prediabetes or diabetes [15]. A multifaceted approach that includes glycemic control along with other vascular risk factor control, such as hypertension and dyslipidemia, is very beneficial for the prevention of macrovascular (cerebral, cardiac, and peripheral vascular) disease [16]. In summary, hypertension and/or diabetes have been strongly associated with an elevated risk of combined vascular events and stroke independent of conventional cardiovascular risk factors. Hypertension is related to a significantly higher combined vascular events and stroke risk than diabetes [17]. Hypertension and diabetes were associated with a significant number of patients with stroke in our study. Many patients who were admitted had uncontrolled blood pressure (most were in stage 2 hypertension). Many patients had uncontrolled blood glucose levels, which was inferred by diabetic range glycosylated hemoglobin level.

Generally, anemia has been associated with a hyperkinetic state, which alters endothelial adhesion molecule genes, leading to thrombus formation. In addition, blood flow augmentation and turbulence may cause the migration of this thrombus, leading to artery-to-artery embolism [18]. Furthermore, neuroimaging of stroke patients with iron deficiency anemia has detected many multifocal and bilateral cerebral infarcts and lesions in both anterior and posterior circulation territories. These findings support the fact that anemic hypoxia causes diffuse ischemic injury in watershed areas of the brain [19]. Furlan et al. found that elevated hemoglobin on the initial admission is associated with more severe strokes, greater disability at discharge, and higher 30-day mortality after acute ischemic stroke. Low hemoglobin on admission is associated with longer stay in the acute care hospital [20]. Dayimu et al. suggested that long-term decreasing hemoglobin levels might increase the risk of stroke [21]. In our study, also, anemia was present in a significant number of patients (63%), which may be due to nutritional deficiency as most of the patients have low socioeconomic status and renal disease.

Ninomiya et al. (2009) in a meta-analysis found that the presence of proteinuria confers about a 70% greater risk of stroke compared to those without it [22]. In a meta-analysis published by Lee et al., microalbuminuria was strongly and independently associated with incident stroke across various population subtypes after adjustment of established cardiovascular risk factors [23]. In this study, also, proteinuria was also present in 39% of patients, which may have been caused by diabetes or hypertension.

Chronic kidney disease (CKD) is strongly associated with the full spectrum of cerebrovascular disease, including ischemic and hemorrhagic stroke, small vessel disease, and vascular cognitive impairment [24]. Dad and Weiner found that chronic kidney disease is an independent risk factor for both hemorrhagic stroke and ischemic stroke subtypes. Patients with end-stage renal disease receiving renal replacement therapy are at fourfold to 10-fold higher risk of stroke as compared to the general population, and stroke risk increases by a factor of sevenfold during the initial year on dialysis [25]. One study published by Go et al. found an independent graded association between renal function and cardiovascular events, where a cardiovascular event was defined as hospitalization for coronary disease, heart failure, stroke, or peripheral arterial disease [26]. Bos et al., in a population-based cohort study, found that decreased glomerular filtration was a strong risk factor for hemorrhagic, but not ischemic, stroke [27]. Lee et al. found out that the incidence of stroke risk increased among participants with an estimated glomerular filtration rate below 60 mL/minute/1.73 m^2^ and not among those with a glomerular filtration rate of 60-90 mL/minute/1.73 m^2^ [28]. The study carried out by Tsagalis et al. found that, in patients hospitalized for first-ever stroke who were admitted for an acute stroke, approximately 28% of acute stroke patients had moderate or severe renal dysfunction, defined as glomerular filtration rate of ≤30 mL/minute/1.73 m^2^ [29]. Abramson et al., in one prospective study, found that stroke risk was increased substantially in patients who had both CKD and anemia independent of other known risk factors [30]. Our study has also shown that most of the patients had stage 2 or stage 3A renal disease, which is also a risk factor for cerebrovascular events as it leads to accelerated atherogenesis and raised blood pressure.

Dyslipidemia was also present in some patients, which is also a risk factor for cerebrovascular events. Dyselectrolytemia has also been present in some cases at the time of admission. Age, hypertension, diabetes, dyslipidemia, and proteinuria were seen in many patients in this study.

In spite of every sincere effort, this study has some limitations. The study sample size was relatively small. This study was conducted at a single center, in a tertiary care hospital, so hospital bias cannot be ruled out. The ongoing COVID-19 pandemic and the lockdown have further hampered the study.

## Conclusions

It is recommended from the present study that screening of hypertension, diabetes, dyslipidemia, and proteinuria in people aged more than 40 years and tight control of already diagnosed cases should be routinely done. Early intervention and management of atrial fibrillation should be done to prevent the incidence of stroke. Heavy alcohol intake and smoking should be discouraged to prevent stroke. There are a large number of people with undetected hypertension and diabetes. Further larger studies need to be performed for a better understanding of risk factors.
